# Research on the stipe cracking of wine-cap mushroom (*Stropharia rugosoannulata*) in different humidity conditions

**DOI:** 10.1038/s41598-023-48608-1

**Published:** 2023-11-30

**Authors:** Lei Huang, Can Si, Hongyu Shi, Chunmei He, Jun Duan

**Affiliations:** 1 Key Laboratory of South China Agricultural Plant Molecular Analysis and Gene Improvement, South China Botanical Garden, Chinese Academy of Sciences, Guangzhou, 510650 Guangdong China; 2 University of Chinese Academy of Sciences, Beijing, 100049 China

**Keywords:** Microbiology, Fungi

## Abstract

*Stropharia rugosoannulata* is a well-renowned edible mushroom due to its nutritional and nutraceutical properties. This article focuses on the study of stipe cracking in *S. rugosoannulata*, a common issue in outdoor cultivation of this mushroom in South China. The findings reveal that the stipe cracks of *S. rugosoannulata* are primarily horizontal (transverse). Typically, cracks appear between the annulus and the middle part of the stipe prior to the opening of the pileus. Following the opening of the pileus, a fresh crack appears on the upper part of the stipe above the annulus. During the growth of *S. rugosoannulata*, two distinct elongation sections are observed in the stipe, separated by the annulus. The location of cracks coincides with these elongation sections, and the sequence of crack occurrences matches with the sequence of these elongation sections. The frequency of stipe cracking varies according to developmental stages and humidity conditions. The conclusion of this study is that *S. rugosoannulata* stipes crack during elongation and within elongation sections when humidity is low (≤ 60%), with the S3 developmental stage having the highest risk of cracking.

## Introduction

*Stropharia rugosoannulata*, also known as Wine-cap mushroom or Roundhead mushroom, has a robust fruiting body. When mature, the stipe grows to a length of 9–15 cm and generates a deeply grooved annulus (ring) after the pileus opens^1^.

*S. rugosoannulata* is not only a food source high in protein, low in fat, and rich in minerals, vitamins, and dietary fiber^2^, but also a high-quality raw material for extracting bioactive compounds such as polysaccharides, sterols, and flavor peptides^3–5^. Additionally, it efficiently degrades and transforms various types of agricultural and forestry waste during its growth process^6^, helping to eliminate environmental pollutants and improve soil fertility^7–9^. *S. rugosoannulata* only needs remarkably simple cultivation techniques, yet it results in high yields^10^. Due to its significant application value in many fields, including dietary nutrition improvement, functional food production, environmental pollution control, ecological agriculture development, agricultural and forestry waste utilization, and rural poverty alleviation, *S. rugosoannulata* is recommended for cultivation by the Food and Agriculture Organization of the United Nations^11^.

*S. rugosoannulata* was first domesticated and cultivated in Germany in 1969. Subsequently, it was introduced and cultivated in other European countries such as Poland, Czechoslovakia, Hungary, and the Netherlands. By 1983, it was introduced to the United States^1, 10^. China introduced a strain of *S. rugosoannulata* from overseas in 1992 and the cultivation of *S. rugosoannulata* began to spread ever since^12^. Since 2013, the cultivation of *S. rugosoannulata* has been vigorously promoted as a poverty-reducing project in various regions of China, leading to a rapid growth in cultivation area and production. In 2021, China’s yield of fresh *S. rugosoannulata* mushroom exceeded 210,000 tons, marking a 43% increase from the yield recorded in 2019^13^, Simultaneously, the cultivation area expanded to over 4000 hectares^14^.

In South China, *S. rugosoannulata* is suitable for outdoor cultivation in winter. It can be cultivated from December to March of the following year^15^. In cultivation practices in Guangdong province, large-scale cracking of *S. rugosoannulata* stipes is often observed, which significantly compromises the integrity of mushroom appearance, thereby diminishing its quality and commercial value.

The cracking of mushroom pileus in response to low humidity is a common phenomenon^1^. Mushrooms with thick and long stipes, such as *Boletus sp., Armillaria sp.*, *Amanita sp.*, *Pleurotus eryngii*, and *S. rugosoannulata*, can also experience stipe cracking in the wild. However, there is limited research on the phenomenon of mushroom cracking, with only reported in *Lentinula edodes* (Shiitake mushroom) the induction of pileus cracking to form flower-like pileus^16^, while studies on stipe cracking have not been reported thus far.

This article aims to investigate the number and location of stipe cracks in *S. rugosoannulata* under different humidity conditions, and to correlate these observations with the elongation process of the stipe during different developmental stages. A diagrammatic model is developed to illustrate stipe cracking, and the mechanisms behind this phenomenon are explained. The article further provides specific humidity conditions that can be applied to the factory cultivation of *S. rugosoannulata* mushrooms.

## Materials and methods

### S. rugosoannulata

*S. rugosoannulata* involved in this research are identified by Professor Jun Duan from the South China Botanical Garden of the Chinese Academy of Sciences as fruiting bodies of the strain “Zhongke No. 1 Red Songrong”, which is a new variety of *S. rugosoannulata* bred through systematic selection by the South China Botanical Garden of the Chinese Academy of Sciences and preserved in the laboratory of this research institution. They were produced by the understory bed cultivation method and the cultivation substrate formulation consisted of 40% rice husk, 40% sawdust, and 20% shredded forest litter. Fallen leaves, with a thickness of approximately 5 to 10 cm, served as the casing layer. The spawn was sowed in early November 2022, and mushrooms were harvested from mid-December 2022 to late March 2023. All methods were carried out in accordance with relevant guidelines.

**Figure 1:**
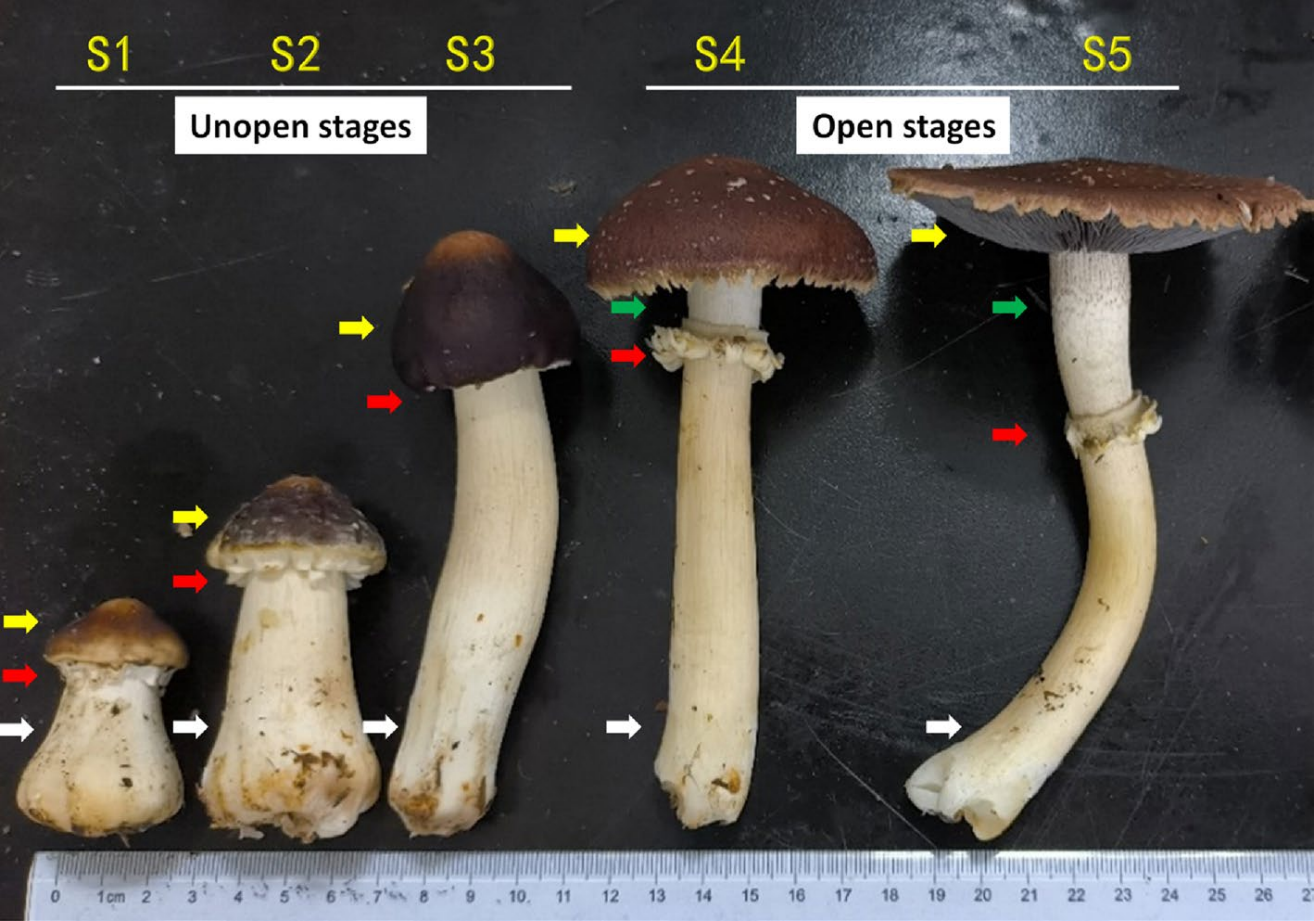
Morphology of *S. rugosoannulata* mushrooms in different developmental stages (red arrows point to the annulus, yellow arrows point to the pileus, white arrows point to the stipe below the annulus, green arrows point to the upper part of stipe above the annulus). S1: young stage; S2: early elongating stage; S3: mid elongating stage; S4: half-open stage; S5: fully open stage.

The growth and development process of *S. rugosoannulata* mushroom can be divided into 5 stages in chronological order^17^. In Stage 1 (S1, young stage), the mushroom has a short and stout stipe. In Stage 2 (S2, early elongating stage), the stipe begins to elongate. In Stage 3 (S3, mid elongating stage), the stipe significantly elongates, but the pileus remains unopen, and no ring structure is visible. In Stage 4 (S4, half-open stage), the pileus starts to open, with the lower edge detaching from the stipe, but it is not fully expanded, taking on a roundhead shape. A ring appears, and the upper part of stipe is exposed. In Stage 5 (S5, fully open stage), the stipe grows to its maximum length and finishes its elongation, and the pileus extends horizontally to the top, releasing spores (Fig. 1).

### Measurement of stipe cracks in *S. rugosoannulata*

Samples of *S. rugosoannulata* mushrooms of all developmental stages with consistent morphology were harvested, with a minimum sample size of 30 individuals per stage. The length of stipes and the location of cracks (the distance from cracks to the base of stipes) were measured. The stipe cracking rate (= number of stipes with cracks / number of stipes × 100%) of each stage were calculated and the maximum number of cracks on each individual stipe were counted. Additionally, relative humidity (RH) and air temperature at the time of harvest were recorded.

### Measurement of stipe elongation in *S. rugosoannulata*

After the young mushrooms emerged on the surface of the cultivation substrate, they were transferred, along with the base substrate, into pots. These pots were then placed indoors, providing a controlled environment with 24 °C and 70% RH, in order to maintain the normal growth of young mushrooms. When the stipe length reached 2.5–3 cm, five young mushrooms with similar growth status were selected to measure the elongation of their stipes using the line-marking method. The initial mushroom pileus (covering the top of stipe internally) was marked as 0, and the initial stipe below the mushroom pileus was divided into 4 equal segments. Horizontal lines were drawn on the stipe using a marker pen, marking them as 1, 2, 3, and 4 from top to bottom. The length of each segment was recorded daily at 10:00 AM for a total of 4 days.

### Measurement of elongation and cracking of the stipes of postharvest* S. rugosoannulata*

Five postharvest *S. rugosoannulata* mushrooms (S2) with consistent growth status were selected. They were placed indoors at 24 °C and three different levels of RH (40%, 60%, 70%). The elongation and cracking of the mushrooms were recorded. The overall length of each mushroom was measured daily at 10:00 AM for a total of 4 days.

### Effects of casing layer on the cracking of *S. rugosoannulata* stipes

A cultivation plot with dense mushroom growth was selected and divided into two equal halves. The casing layer of one half was removed, exposing the stipes of *S. rugosoannulata* mushrooms completely to the air. In the other half, the thickness of the casing layer was increased to 15 cm, burying the mushrooms completely within the casing layer. On both of the aforementioned plots, ten individuals in consistent growth status from various developmental stages were selected, and the cracking of stipes was recorded one day after. The experiment was conducted separately on three different dates at RH of 40%, 55%, and 70%. The cracking rate of *S. rugosoannulata* stipes in different developmental stages was calculated, and the RH inside the casing layer was recorded.

### Statistical analysis

Statistical analyses were performed using Excel 2013. Data with repeats were presented as the mean ± standard deviation. The illustration diagram of stipe cracks and elongation process were created by Microsoft Office 2013.

## Results

### Morphology of cracks on *S. rugosoannulata* stipes

**Figure 2:**
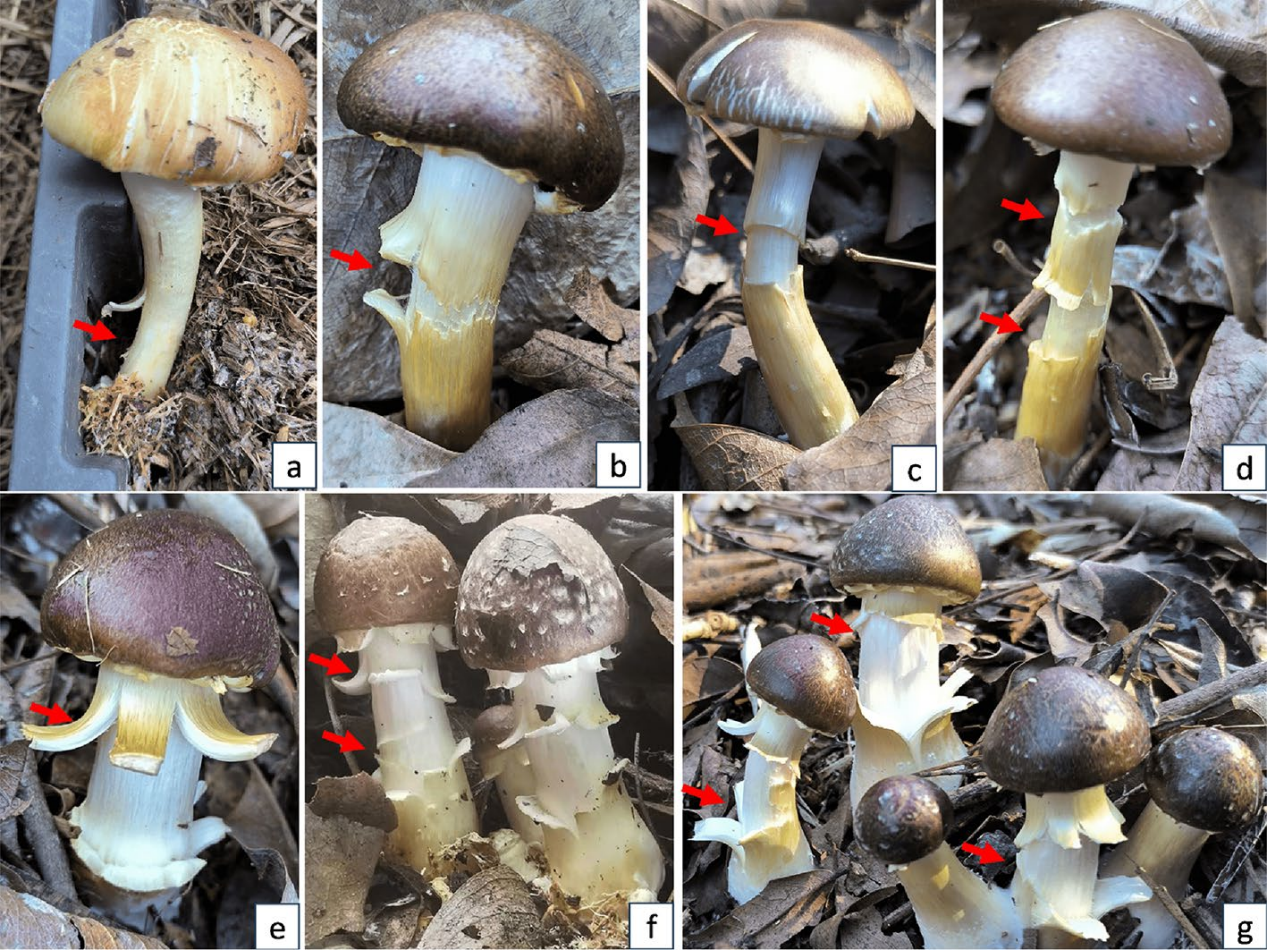
Transverse and longitudinal cracks on *S. rugosoannulata* stipes (red arrows point to the cracks, (**a**)–(**d**) transversec cracks, (**e**)–(**g**) longitudinal cracks).

During the development of *S. rugosoannulata* mushrooms, it is common for stipes to experience cracking (Fig. 2). The main type of cracks observed on the stipes of *S. rugosoannulata* is horizontal (transverse). Some horizontal cracks can encircle the stipe, forming circular cracks (Fig. 2c,d), while others may only occur on one side of the stipe (Fig. 2a,b). In certain cases, transverse cracks on the mushroom stipes may give rise to multiple longitudinal cracked pieces that outwardly curl. These segments are arranged in a radial pattern with two distinct levels (Fig. 2e–g). Some stipes may exhibit two parallel cracks at different heights (Fig. 2d). In other cases, new cracks may occur within an existing crack, resulting in overlapping positions of the two cracks (Fig. 2f). The outer layer of the cracked portion of the stipe appears harder and darker in color, while the interior of the cracks and the uncracked portions have a softer texture and a lighter color (Fig. 2f).

### Location and number of cracks on *S. rugosoannulata* stipes

**Figure 3:**
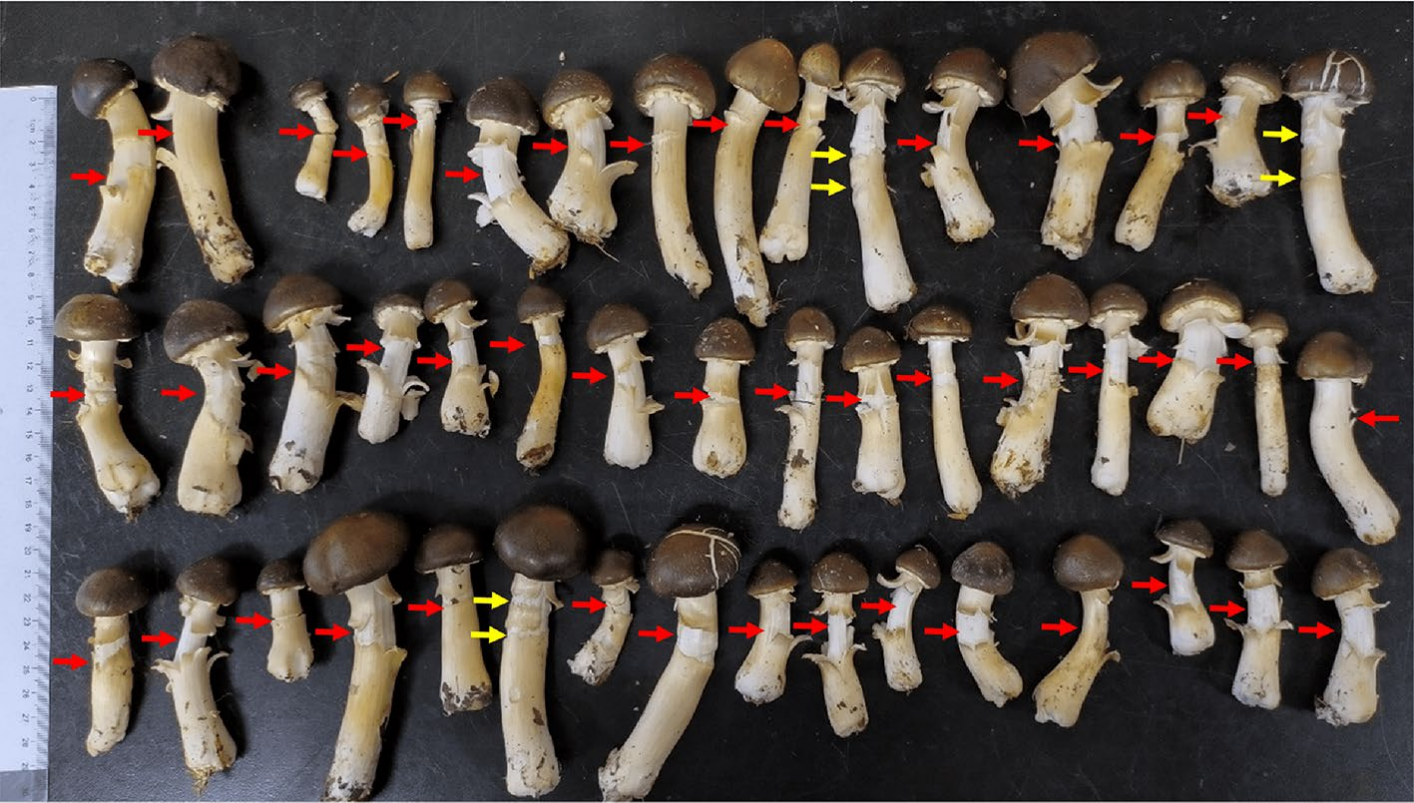
Cracks on *S. rugosoannulata* stipes in unopen stages (red arrows point to the only one crack, yellow arrows point to the two parallel cracks).

**Figure 4:**
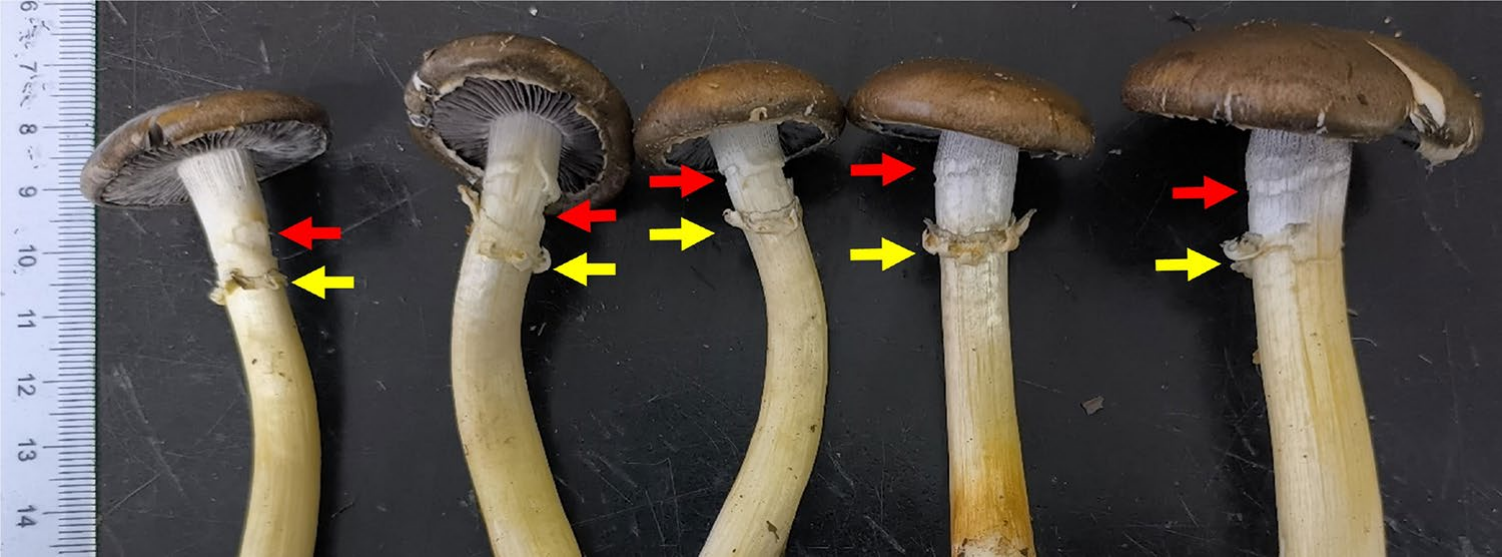
Cracks on *S. rugosoannulata* stipes in the open stages (red arrows point to the cracks, yellow arrows point to the annulus).

The location where cracks occur on the stipe of *S. rugosoannulata* mushrooms is typically on the portion of the stipe that extends beyond the casing layer and exposes to the air (Fig. 2). At the unopen stages, the cracks typically occur in the upper-middle portion of the stipe below the annulus. The distance from the crack to the base of the stipe ranges from 3.0 to 9.0 cm. The maximum number of cracks is two, and they are parallel with a distance of approximately 1.0 to 2.0 cm (Fig. 3). At the open stages, there may also be one crack on the upper part of stipe, approximately 0.5 to 1.5 cm above the annulus (Fig. 4). The number of transverse cracks on an individual stipe range from one to two.

### Cracks on *S. rugosoannulata* stipes in different developmental stages

**Figure 5:**
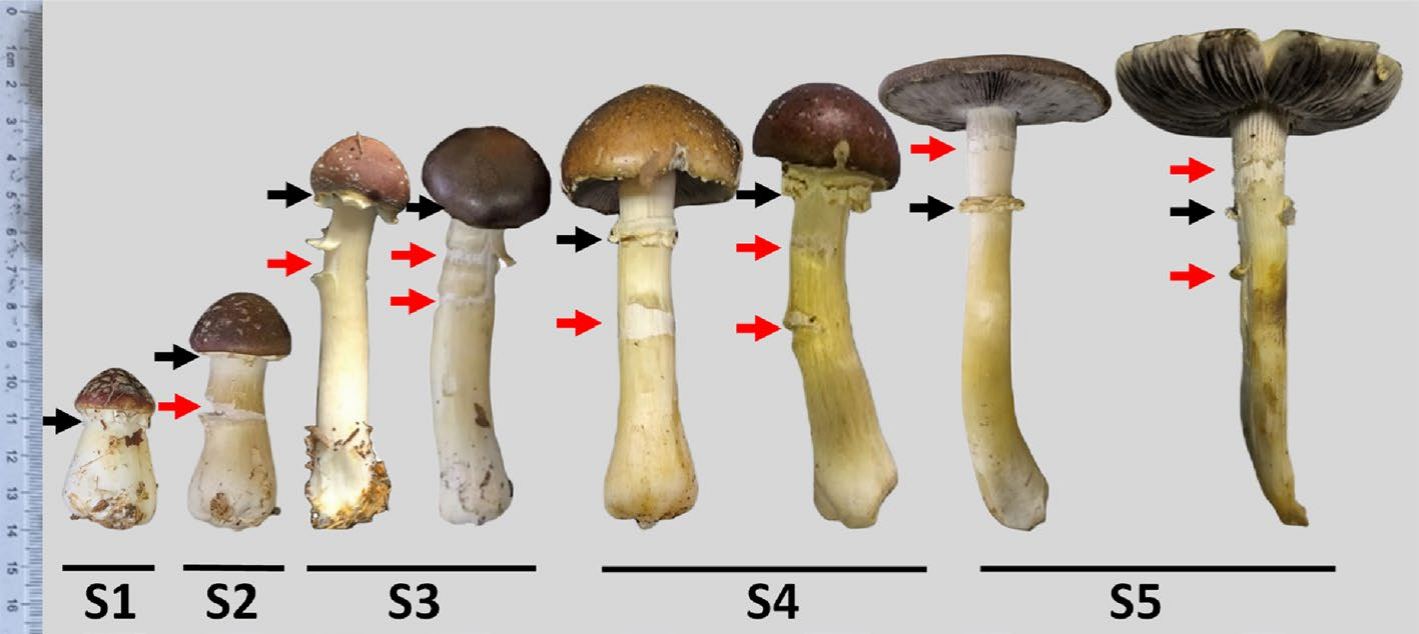
Cracks on *S. rugosoannulata* stipes in different developmental stages (red arrows point to the cracks, black arrows point to the annulus, S1–S5: developmental stages in in chronological order).

During the development process of *S. rugosoannulata*, the stipes elongated by nearly 9 cm (Table 1). In S1, no cracks were observed on the stipes. However, in the stages from S2 to S5, cracks occurred on the stipes. In S2, the maximum number of cracks observed was one. In S3, the number of cracks could increase by one, with a maximum of two cracks. These cracks were located below the annulus. The occurrence of cracks on the stipes in S4 was similar to S3. In S5, the stipe may crack once above the annulus, but only on stipes that have experienced a maximum of 1 crack below the annulus. In S4 and S5, the maximum number of cracks observed on a stipe was two (Fig. 5, Table 1).

**Table 1:** Comparison of cracks on *S. rugosoannulata* stipes in different developmental stages.

Developmental stages	S1	S2	S3	S4	S5
Length of mushrooms/cm	3.9 ± 0.3	5.8 ± 0.5	10.6 ± 1.0	11.5 ± 1.2	12.6 ± 1.3
Location of annulus/cm	2.4 ± 0.2	4.0 ± 0.3	9.1 ± 0.9	9.2 ± 0.9	9.4 ± 0.9
Location of cracks/cm	–	3.1 ± 0.3	7.3 ± 0.6 5.3 ± 0.5	7.2 ± 0.5 5.0 ± 0.5	10.5 ± 1.0 5.0 ± 0.5
Maximum number of cracks	0	1	2	2	2

- means no existence; location means the distance to the base of stipe.

**Figure 6:**
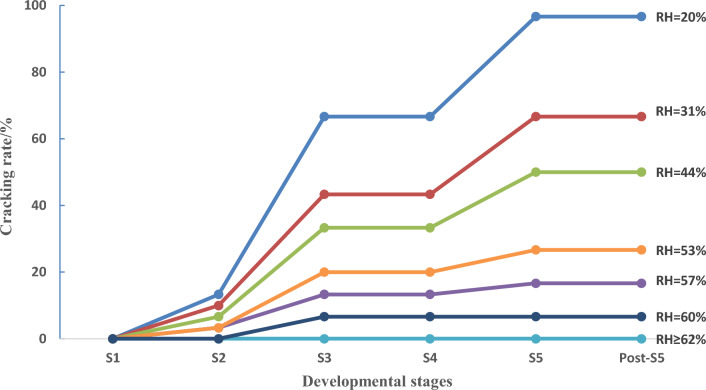
Cracking rate of *S. rugosoannulata* stipes in different developmental stages and humidity conditions (RH: relative humidity).

The cracking characteristics of *S. rugosoannulata* stipes are summarized as follows: Stipe cracking was observed to start from S2, but the cracking rate was lowest in this stage. As the stipes elongated, the cracking rate increased, reaching its maximum in S5. After S5, the cracking rate remained stable. There was a significant increase in the cracking rate during the transitions from S2 to S3 and from S4 to S5, and the increase during S2 to S3 transition was greater. Among all the stages, S3 had the highest likelihood of stipe cracking (Fig. 6). Meanwhile, S3 is also the optimal harvesting stage for *S. rugosoannulata* mushrooms as they approach commercial maturity in this stage^17^. This indicates that stipe cracking has a significant destruction to the commercial value of mushrooms.

### Environmental conditions influencing the cracking of *S. rugosoannulata* stipe

**Figure 7:**
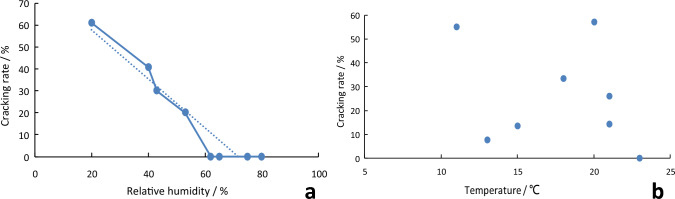
Environmental conditions influencing the cracking of *S. rugosoannulata* stipes (the blue dashed line represents the trend line, (**a**) RH, (**b**) temperature). Note: Data comes from *S. rugosoannulata* of S3.

When RH of the cultivation field was ≤ 60%, cracks occurred on *S. rugosoannulata* stipes. When the RH dropped to around 20%, the cracking rate of the stipes reached nearly 100%. Conversely, when the RH of the cultivation field was ≥ 62%, cracks was not observed on *S. rugosoannulata* stipes (Fig. 6). RH of the cultivation field showed a negative correlation with the cracking rate of *S. rugosoannulata* stipes (Fig. 7a). Stipe cracking occurred in a temperature range of 11–23 °C, and there was no significant correlation between the cracking rate and temperature of the cultivation field (Fig. 7b).

### Elongation process of *S. rugosoannulata* stipes

**Figure 8:**
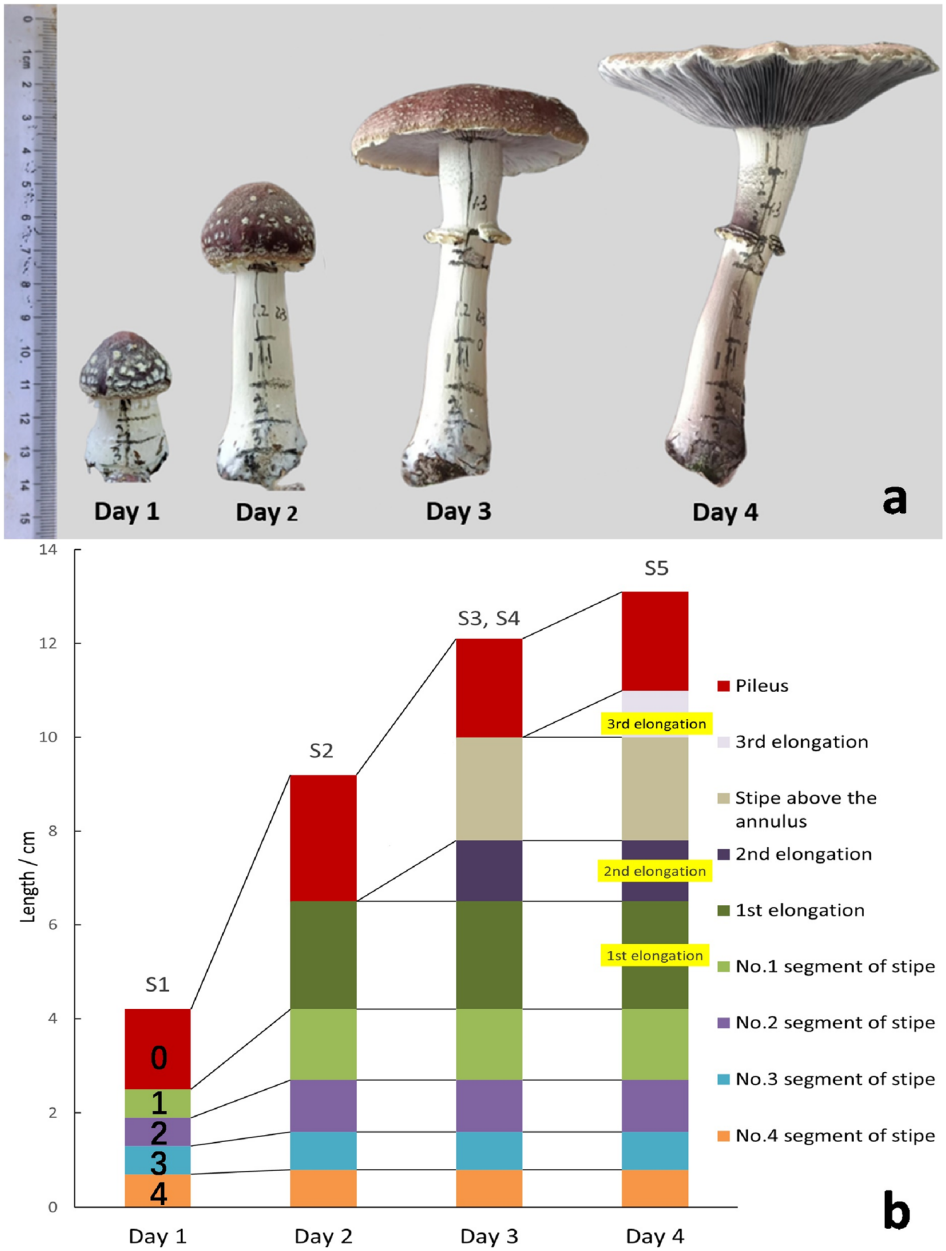
Photoes and illustration diagram of the elongation process of *S. rugosoannulata* stipe ((**a**) photoes, (**b**) illustration diagram; 24 °C, 70% RH; 0 means initial pileus, 1–4 means four segments of initial stipe).

When cultivated at 24 °C and 70% RH, *S. rugosoannulata* stipes increased from an average height of 4.2 cm to 13.1 cm within a period of four days. And after the fourth day, the stipe no longer elongated (Fig. 8a). Each segment marked from 0 to 4 exhibited elongation, but the elongation of *S. rugosoannulata* stipe primarily occurred in the upper-middle portion of the stipe, especially in the portion covered by the pileus (segment 0) (Table 2).

**Table 2:** Comparison of elongation of different segments of *S. rugosoannulata* stipe*.*

Marking number of stipe segments	0	1	2	3	4	Whole mushroom
Initial length/cm	1.7 ± 0.2	0.6 ± 0.1	0.6 ± 0.1	0.6 ± 0.1	0.7 ± 0.1	4.2 ± 0.3
Final length/cm	8.9 ± 0.5	1.5 ± 0.2	1.1 ± 0.1	0.8 ± 0.1	0.8 ± 0.1	13.1 ± 0.8
Length of elongation/cm	7.2 ± 0.5	0.9 ± 0.2	0.5 ± 0.1	0.2 ± 0.1	0.1 ± 0.0	8.9 ± 0.6
Portion of segment elongation/%	80.9	10.1	5.6	2.2	1.1	100

When cultivated at 24 °C and 70% RH, the stipe inside the pileus experienced three instances of elongation over a period of three days, with daily increments of 2.3 cm, 1.3 cm, and 1.0 cm respectively. Three elongation sections were located at specific distances from the base of the stipe: 4.2–6.5 cm, 6.5–7.8 cm, and 10.0–11.0 cm. Elongation process of the stipe was divided into two periods based on the occurrence of the annulus (the opening of the pileus). Before the annulus appeared, the stipe underwent elongation below it, resulting in the formation of the first and second elongation sections. These two sections were connected, forming a continuous elongation section. After the annulus appeared, the stipe below the annulus stopped elongating, while the upper part of stipe above the annulus continued to elongate, resulting in the occurrence of another elongation section at the top of the stipe. The elongation section of the stipe after the pileus opening is separated from the elongation section before the pileus opening by the annulus, creating two independent elongation sections of the stipe (Fig. 8b).

By comparing the locations where *S. rugosoannulata* stipes cracked and elongated, it can be discovered that these two locations largely coincide with each other, both in specific section and in chronological order (Figs. 5 and 8b).

### Elongation and cracking of postharvest *S. rugosoannulata* stipes

**Figure 9:**
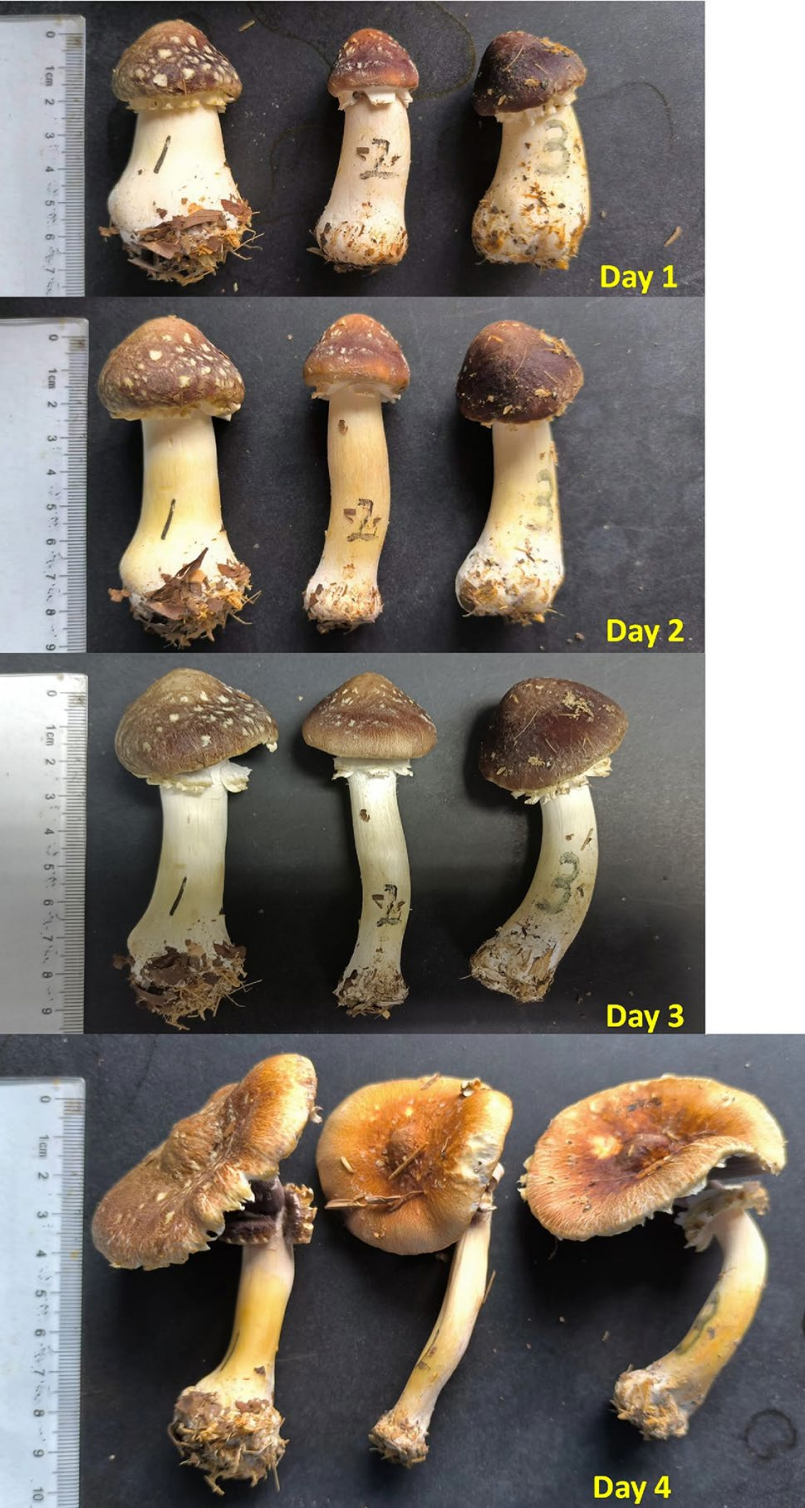
Elongation of postharvest *S. rugosoannulata* stipes (24 °C, 60% RH).

Postharvest *S. rugosoannulata* mushrooms were still capable of completing the growth and development process from S2 to S5 (Fig. 9), with the stipe undergoing a noticeable elongation of 3.2 cm. However, during the entire elongating process, the stipes did not exhibit any cracking when the RH was low (40–60%) (Table 3). This suggests that maintaining a connection between the stipe and the substrate was a necessary condition for stipes to crack.

**Table 3:** Elongation and cracking rate of postharvest *S. rugosoannulata* stipes.

Days postharvest	Developmental stages	Length of mushroom/cm	Cracking rate/%
Day 1	S2	6.7 ± 0.3	0
Day 2	S3	8.7 ± 0.4	0
Day 3	S4	9.3 ± 0.4	0
Day 4	S5	9.9 ± 0.4	0

The data of length was measured at 24 °C and 60% RH.

### Effect of casing layer on stipe cracking in *S. rugosoannulata*

After the casing layer was removed, *S. rugosoannulata* stipes were exposed to the air. When the RH was 40% and 55%, mushrooms in the initial stages of S1, S2, S3 and S4 exhibited stipe cracking after one day of growth, while mushrooms in the initial stage of S5 did not show any stipe cracking. Conversely, when a thicker casing layer completely covered all the mushrooms, RH inside the casing layer increased to a range of 63% to 75%, significantly higher than the RH outside. In this situation, none of the *S. rugosoannulata* stipes experienced any cracking (Table 4). These results indicated that *S. rugosoannulata* stipe only cracks in the elongation stages, particularly when the RH is low. Once the elongation stopped, the stipe no longer cracks. Casing layer effectively increases the environmental humidity and prevents stipe cracking of *S. rugosoannulata*.

**Table 4:** Cracking rate of *S. rugosoannulata* stipes in different developmental stages with or without casing layer.

Initial stage	Treatment					
	Substrate without casing layer			Substrate with 15 cm-thick casing layer		
	40% RH	55% RH	70% RH	40% RH	55% RH	70% RH
S1	30.00	10.00	0.00	0.00	0.00	0.00
S2	90.00	50.00	0.00	0.00	0.00	0.00
S3	40.00	30.00	0.00	0.00	0.00	0.00
S4	60.00	30.00	0.00	0.00	0.00	0.00
S5	0.00	0.00	0.00	0.00	0.00	0.00

*RH* means relative humidity.

**Figure 10:**
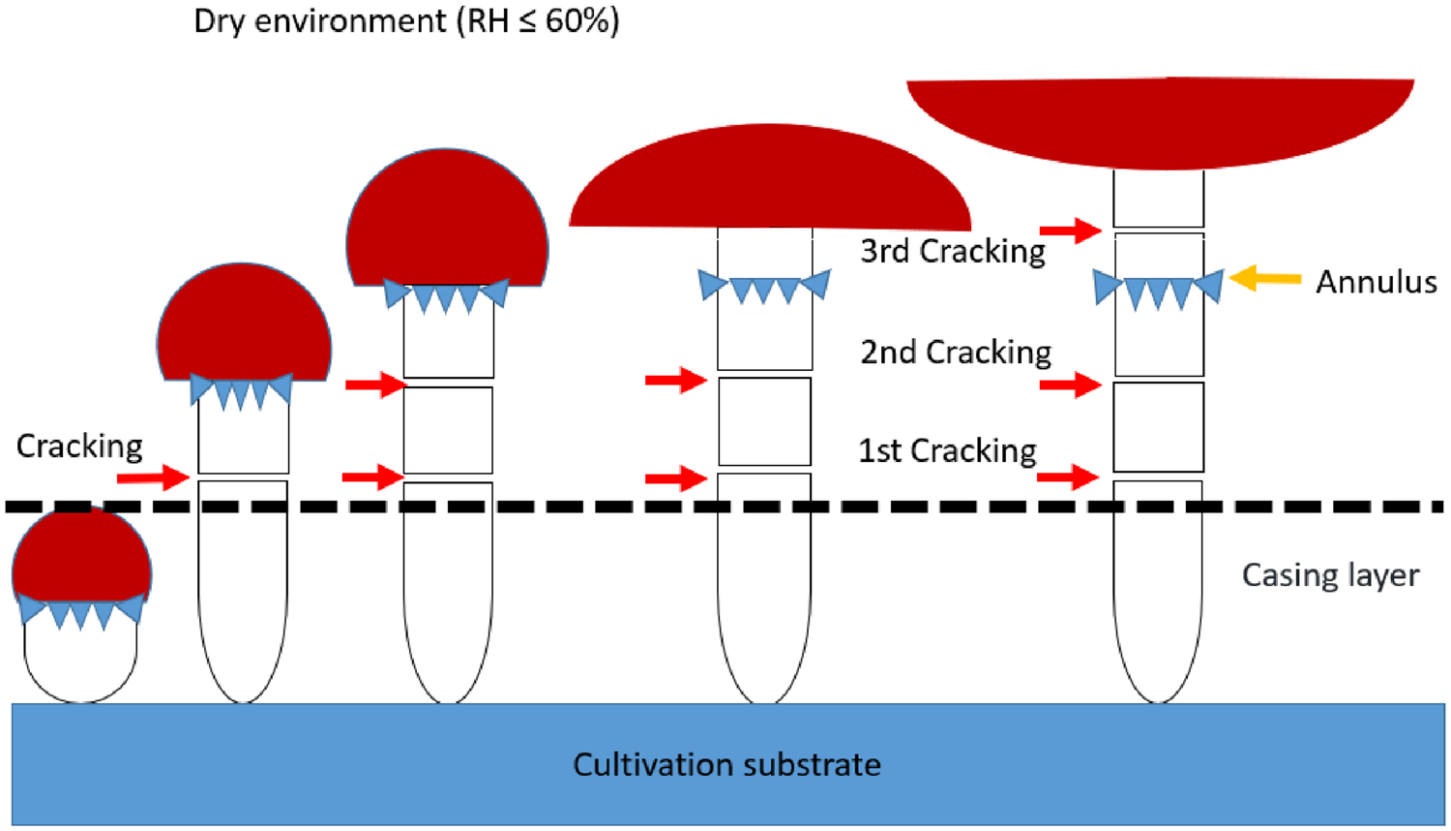
Model of cracking on the elongating stipe of *S. rugosoannulata* (RH: relative humidity).

In summary, *S. rugosoannulata* stipe is probable to crack (mainly in the form of horizontal cracks) during the process of elongation when environmental RH is ≤ 60%. There are three locations where the stipe cracks: two are located in the upper-middle part of stipe below the annulus, and one is located in the upper part of stipe above the annulus. These cracking locations generally coincide with the elongation section of the stipe. Before the pileus opens, the stipe below the annulus elongates, and there may be one to two instances of cracking in the elongation section. After the pileus opens, the stipe below the annulus stops elongating, while the stipe above the annulus continues to elongate, and there may be one instance of cracking in the elongation section. Stipes that have experienced cracking twice below the annulus do not crack again above the annulus. Once the stipe has stopped elongating, no further cracking occurs, and postharvest *S. rugosoannulata* stipes do not exhibit any cracking. Increasing environmental humidity can effectively prevent stipe cracking. The diagram illustrating stipe cracking of *S. rugosoannulata* is shown in Fig. 10.

## Discussion

### The mechanism of stipe cracking in *S. rugosoannulata*

Turgor pressure is the pressure exerted by water-filled vacuoles against the cell wall, resulting in an outward force on the cell wall. Turgor pressure creates mechanical stress within the cell wall^18^. Mushroom maintains turgor pressure by absorbing water from the substrate through mycelium, which is one of the driving forces for stipe elongation. When mushroom cells absorb water, the turgor pressure increases^19^. In low RH (≤ 60%) environments, on one hand, the outer layer of the stipe undergoes dehydration and shrinks, becoming dry and rigid, probably forming micro-cracks^20^. On the other hand, as turgor pressure increases within the stipe, the stress exerted on the outer layer of the stipe also increases, causing the micro-cracks to widen and eventually develop into visibly noticeable cracks. The reason for the occurrence of two parallel cracks on the stipe may be related to the turgor pressure changing in the form of pulses during mycelial growth^21^. After being harvested, *S. rugosoannulata* no longer receives water supply from substrate, resulting in a decrease in turgor pressure within the stipe. As a result, cracking does not occur during the elongation process. Therefore, the external cause contributing to stipe cracking in *S. rugosoannulata* is mainly the low-humidity environment, while the internal cause is primarily the turgor pressure within the stipe.

### Less cracks in reality than in theory

According to the model in Fig. 10, a fully developed stipe of *S. rugosoannulata* has three potential cracking locations. Theoretically, all three locations may crack, resulting in a maximum of three cracks. However, the results of this study indicate that on a single stipe, it is unlikely to observe horizontal cracks in all three potential cracking locations. Out of the three possible cracking locaitons, a maximum of two locations may exhibit cracking simultaneously, with a maximum of two instances of cracking occurring. Stipes that have experienced two instances of cracking below the annulus will not experience further cracking above the annulus. The most common occurrence is a single instance of cracking in one specific location of stipe below the annulus. The reason for the actual number of stipe cracking being lower than the theoretical number may be related to the release of turgor pressure within the stipe after the previous cracking occurs^22^. If the stipe has already experienced two instances of cracking, most of the turgor pressure within the stipe will be released, and the remaining pressure will not be sufficient to cause further cracking on the stipe.

### Lack of humidity management leading to stipe cracking in *S. rugosoannulata*

In South China, traditional outdoor cultivation is still the major way to produce *S. rugosoannulata* mushroom. This way of cultivation cannot avoid the limitations in humidity management due to less sophisticated infrastructure and environmental control, unlike the way of factory cultivation in which humidity can often be precisely controlled through advanced instruments^11^. This research proves again that humidity management is essential in the production of high-quality mushrooms.

To meet the temperature requirements (10–25 °C) for the growth and development of *S. rugosoannulata*, the cultivation seasons for most regions outside Guangdong are primarily spring and autumn. These seasons typically have higher rainfall^23^. In contrast, *S. rugosoannulata* is cultivated in Guangdong during the winter, which experiences less rainfall^24^. Taking Guangzhou as an example, the outdoor cultivation of *S. rugosoannulata* in Guangzhou occurs from December to March, which is the period with the lowest rainfall in the year. The monthly rainfall during this time is generally below 100 mm, with December and January experiencing less than 30 mm of rainfall. This period is characterized by a winter drought that occurs consistently throughout the year^25–27^. Dry environment is an external cause that contributes to stipe cracking in *S. rugosoannulata*. In regions outside of Guangdong, the cultivation seasons for *S. rugosoannulata* typically have a relatively humid environment, which seldom reaches the low level of humidity required for stipe cracking to occur. As a result, the phenomenon of stipe cracking may be less common in these regions. However, the entire cultivation season of *S. rugosoannulata* in Guangdong meets a dry and low rainfall climate. This leads to the widespread occurrence of stipe cracking in this region.

## Data Availability

The authors confirm that all data generated or analysed during this study are included in this published article.

## References

[1] Stamets, P. & Chilton, J. S. *The Mushroom Cultivator: A Practical Guide to Growing Mushrooms at Home*. http://library.uniteddiversity.coop/Permaculture/Mushroom Cultivator-A Practical Guide to Growing Mushrooms at Home.pdf. (1983).

[2] Jing B-N (2022). Analysis and evaluation of nutrient components, bioactive substances and heavy metal content of *Stropharia rugosoannulata* in Bo’ai County. Sci. Technol. Food Ind..

[3] Li X-X (2023). Three-phase extraction of polysaccharide from *Stropharia rugosoannulata*: Process optimization, structural characterization and bioactivities. Front. Immunol..

[4] Wu J (2013). Isolation of bioactive steroids from the *Stropharia rugosoannulata* mushroom and absolute configuration of strophasterol. Biosci. Biotechnol. Biochem..

[5] Chen W-C (2022). Characterization of novel umami-active peptides from *Stropharia rugoso-annulata* mushroom and in silico study on action mechanism. J. Food Compos. Anal..

[6] Kamra DN, Zadražil F (1985). Influence of oxygen and carbon dioxide on lignin degradation in solid state fermentation of wheat straw with *Stropharia rugosoannulata*. Biotechnol. Lett..

[7] Castellet-rovira F (2017). *Stropharia rugosoannulata* and *Gymnopilus luteofolius*: Promising fungal species for pharmaceutical biodegradation in contaminated water. J. Environ. Manag..

[8] Pozdnyakova N (2018). The degradative activity and adaptation potential of the litter-decomposing fungus *Stropharia rugosoannulata*. World J. Microbiol. Biotechnol..

[9] Gong S (2018). Effects of wine-cap *Stropharia* cultivation on soil nutrients and bacterial communities in forestlands of northern China. Peerj.

[10] Szudyga K (1978). Stropharia rugoso-annulata: The Biology and Cultivation of Edible Mushrooms.

[11] Marshall E, Nair NG (2009). Make money by growing mushrooms.

[12] Luo Q (2022). Biological basis and bioactive components of *Stropharia rugosoannulata* and its application. Microbiol. China.

[13] China Edible Fungi Association (2023). Statistical survey of edible fungi in China in 2021. Edible Fungi China.

[14] Xiong W-Q (2021). Cultivation status and development recommendations of Stropharia rugosoannulata. Edible Fungi.

[15] Ma L-F (2021). Effects of different *Stropharia rugosoannulata* culture medium on growth, yield and quality. North. Hortic..

[16] Wang C-C (2023). Study on the grade evaluation method based on the pileus characteristics offlower *Lentinula edodes* mushroom and suitable environmental conditions for its formation. Edible Med. Mushrooms.

[17] Chen R-R (2022). Nutrients, texture and taste characteristics of *Stropharia rugosoannulata* during growth and development. Acta Edulis Fungi.

[18] Ortega JKE (2023). Theoretical analyses of turgor pressure during stress relaxation and water uptake, and after changes in expansive growth rate when water uptake is normal and reduced. Plants.

[19] Kamada T (1994). Stipe Elongation in Fruit Bodies.

[20] Benzahar HH (2020). Influence of Micro-Crack on the Propagation of a Semi-Infinite Crack.

[21] Johns S (1999). Pulses in turgor pressure and water potential: Resolving the mechanics of hyphal growth. Microbiol. Res..

[22] Galstyan A, Hay A (2018). Snap, crack and pop of explosive fruit. Curr. Opin. Genet. Dev..

[23] Yao S-B (2017). Seasonality of precipitation over China. Chin. J. Atmos. Sci..

[24] Guo J (2008). Dynamic Monitoring and Quantitative Evaluation of Drought in Guangdon, China.

[25] Guangzhou Meteorological Bureau. *Guangzhou Climate Bulletin, 2020* (2021).

[26] Guangzhou Meteorological Bureau. *Guangzhou Climate Bulletin, 2021* (2022).

[27] Guangzhou Meteorological Bureau. *Guangzhou Climate Bulletin, 2022* (2023).

